# Rare Presentation of Dercum’s Disease in a Child with Abnormalities in Lipoprotein Metabolism

**DOI:** 10.5935/abc.20180191

**Published:** 2018-11

**Authors:** Maria Cristina de Oliveira Izar, Henrique Andrade Rodrigues da Fonseca, Carolina Nunes França, Valéria Arruda Machado, Carlos Eduardo dos Santos Ferreira, Francisco Antonio Helfenstein Fonseca

**Affiliations:** Escola Paulista de Medicina - Universidade Federal de São Paulo, São Paulo, SP - Brazil

**Keywords:** Adiposis Dolorosa, Rare Diseases, Inflammation, Lipid Metabolism Disorders, Child, Dyslipidemias

*Adiposis dolorosa*, or Dercum’s disease, is a subcutaneous accumulation of fat in the body accompanied by intense, chronic, and symmetrical pain, often disabling, and usually not responsive to conventional analgesics. It was first described by Dercum, recognized as a separate disease in 1892,^1^ and further reported by White in 1899.^2^ Termed in the literature Dercum’s disease, Morbus Dercum, *lipomatosis dolorosa*, adiposalgia, *adiposis dolorosa*, and adipose tissue rheumatism, this condition is more prevalent in young women, aged 35 to 50 years, and affects preferably those in the post-menopause phase.^1^^-^^3^*Adiposis dolorosa* can also occur in multiple familial lipomatosis, a condition associated with multiple lipomas.^4^ Other symptoms and signs include psychiatric (depression, anxiety, sleep disturbances, memory and concentration impairment), cardiovascular (tachycardia), pulmonary (shortness of breath), rheumatological (fatigue, weakness, joint and muscle aches) and gastrointestinal (bloating, constipation) disorders.^3^

Dercum’s disease was described as a general disease of the lymphatic system. In 2014, Rasmussen et al.^5^ suggested that this is a lymphovascular disorder with abnormalities in the adipose tissue deposition and lymphatic transport, showing that lipomas appeared to be fed and drained by functional lymphatics. In addition, Huang et al.^6^ have reinforced the importance of lymphatic system in cholesterol transport, showing the association with ApoA1, HDL formation, and lymphatic transport to the blood for scavenging by the HDL receptor, or scavenger receptor B1.^6^

Although the majority of Dercum’s disease cases occurs sporadically, there are reports suggesting an autosomal dominant inheritance, with variable expression. The prevalence and the pathophysiology are also unknown, but inflammation, endocrine, adipose tissue, and nervous system dysfunction, trauma, mechanical pressure on the nerves, are possible etiological conditions.^3^^-^^4^ Considering the abnormal fat deposition, presence of inflammation, and possible metabolic and lipoprotein abnormalities, an increased risk for atherosclerosis should be expected. Albeit the increased fat mass accumulation in Dercum’s disease, it has not been yet reported in association with cardiovascular diseases.^7^

Dercum’s disease seems to be rare in children, as the disease usually manifests in adulthood. In the present study, we report the rare case of a child with Dercum’s disease associated with presence of marked dyslipidemia and inflammation.

**Figure 1:**
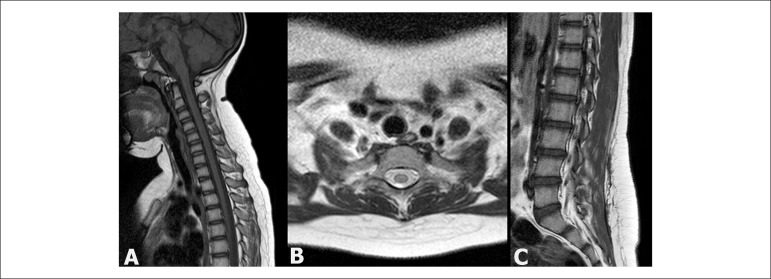
Magnetic resonance images acquired in the A) sagital plane (T1-weighted) and in the B) axial plane (T2-weighted) showing diffusely prominent subcutaneous adipose tissue without delineation of margins or signs of an encapsulated lesion. C) Similar findings are observed in the lumbar region on the T1-weighted sagital image, where it is also possible to identify a linear scar, secondary to a previous surgical resection.

An eight-year-old female child presented with lipomatosis in the backbone, with pain, who became resistant to standard pain-relief medications within one year. Magnetic resonance imaging (MRI) of the backbone revealed the presence of multiple diffuse lipomas (Figure 1), reinforcing the suspicion of Dercum’s disease.^1^^,^^2^ Many surgical procedures were performed to remove those lipomas, but abnormal fat deposition and pain progressed over time, with impairment of daily activities, requiring combined analgesic medication, including morphine. Lipomas increased in number and size, affecting the backbone, legs, arms, face, neck, and abdominal wall. Fat deposition also included liver steatosis, confirmed by MRI. The patient is currently 13 years-old with sexual maturity range II (by Tanner staging).

It is believed that this is a variant presentation of Dercum’s disease, first classified as a localized nodular form that further became generalized and affected a prepubescent girl. This diagnosis was confirmed after ruling out other pathologies with similar clinical presentation, such as those described by Hansson et al.^3^ in 2012.

There were no reports of lipomatosis in any other family member, including parents and siblings.

Laboratory analyses before therapy, to appraise glucose metabolism, lipids and genetic factors revealed hyperinsulinemia (31.8 uU/ml), with normal fasting glucose levels (81 mg/dl) and HbA1c (3.8%), at baseline. Fasting lipid analyses showed low HDL-c (19.3 mg/dl) and Apo A1 (112 mg/dl) concentrations, hypertriglyceridemia (320 mg/dl), hyperbetalipoproteinemia (118 mg/dl), LDL-c in the normal range (108 mg/dl), but with increase in small dense LDL particles (> 40 mg/dl). Her HDL map showed high pre-beta HDL (29 mg/dl; normal < 17 mg/dl), normal alpha 4 HDL (normal< 5.3 mg/dL), high HDL-3 (33mg/dl; normal < 13.5 mg/dl), low HDL 2 (19.3 mg/dl; normal > 45 mg/dl) and HDL-1 (9.5 mg/dl; normal > 29.3 mg/dl), thus showing the incapacity of larger HDL particles formation, with an excess of smaller, less protective particles.

Cholesterol synthesis marker (lathosterol) was below detection level, whereas beta-sitosterol/cholesterol and campesterol/cholesterol ratios were 115 and 149 µmol/mmol of cholesterol (in the normal range). Inflammatory markers, such as high sensitivity-C-reactive protein (13.8 mg/L) and lipoprotein-associated phospholipase A2 (Lp-PLA2, 375ng/ml), were very high.

The child did not present signs of thyroid dysfunction. Sexual and intermediary hormones androstenedione (219 ng/mL), 17-hidroxiprogesterone (76 ng/dL), testosterone (124 ng/dL), and estradiol (24.10 pg/mL) were high for her age. Dehydroepiandrosterone-sulphate (28.4 µg/dL) and growth hormone (0.67 ng/mL) were in the normal range.

Normal concentration of N-terminal pro-B-type natriuretic peptide (NT pro-BNP) was observed, reflecting no myocardial dysfunction.

Genetic analysis showed apolipoprotein E genotype E3/E4 and Factor V Leiden -/-, not representing genetic risk factors for cardiovascular disease.

Body composition was evaluated via bioelectrical impedance analysis (BIA 450, Biodynamics Inc, USA), revealing normal levels of water in the body (23.1 L), but high fat mass component (40%), for gender and age.

The therapeutic regimen adopted for the child was metformin 850 mg, atorvastatin 20 mg, losartan 25 mg, hydrochlorothiazide 12,5 mg, gabapentin 300 mg three times a day, fentanyl adhesive 12.5 mcg every 72 h, amitriptyline 50 mg at night for reduction of the neuropathic pain, and morphine 10 mg in exceptional pain crises.

To our knowledge, this is the first report of a case of Dercum’s disease affecting a prepubescent child with lipomas in the dorsal region, face and neck, abdominal wall, arms and legs, which are common sites for lipomas seen in patients with Dercum’s disease in adulthood.^7^ The presentation of lipomas in the backbone can produce a compression of the neural plexus, causing extreme pain, extending to upper and lower limbs, and anterior upper trunk, thus limiting normal daily activities.

The child features a rare presentation of Dercum's disease or *lipomatosis dolorosa* at a young age. The diagnosis of Dercum’s disease was based on the differential diagnosis with other lipomatosis, as recently proposed by Hansson et al.^3^

Her parents and siblings did not show any signs of lipomatosis or *lipomatosis dolorosa*, ruling out the diagnosis of familial multiple lipomatosis, as described by Campen et al.^4^

Besides the abnormal fat accumulation, interesting findings observed in the patient were hyperinsulinemia, low HDL-cholesterol, hyperbetalipoprotinemia, with predominance of small-dense LDL and HDL particles, characterizing an insulin resistance state. The HDL map revealed a phenotype of particles associated with increased cardiovascular risk, with low concentration of HDL-2 particles, which are related to cardiovascular protection, and high concentration of the less protective HDL-3 particles.^8^ These changes in the HDL particle profile can occur by inherited and acquired factors, or secondary to drugs and vigorous aerobic exercise, and in chronic high alcohol intake. The predominance of small dense LDL is associated with progression of atherosclerosis and is frequent in subjects with multiple risk factors for cardiovascular disease, such as diabetes, obesity, and other insulin resistance states.^9^ In patients with lipomatosis, an association with altered activity of lipoprotein lipase (LPL) in the lipomatous tissue, affecting the metabolism of HDL particles, has been also described.^10^ However, the authors do not regard LPL activity as the most acceptable mechanism to justify the changes in lipoprotein sub-fractions observed in Dercum’s disease. The present study did not assess LPL activity in this patient, and other mechanisms may have affected the remodeling of lipoprotein sub-fractions.

High concentration of inflammatory markers, such as lipoprotein associated phospholipase A2 (Lp-PLA2) and C-reactive protein, are in accordance with a pro-inflammatory state that accompanies these lipomas. Also, small dense LDL particles can interact with Lp-PLA2, thus contributing for the synthesis of products that start the inflammatory signaling cascade by C-reactive protein.^11^

The normal synthesis and absorption of cholesterol, as well as thyroid hormone secretion and Apo E genotype, cannot explain the genesis of these lipomas. It is possible that changes in glucose metabolism in the lipomas, imbalance between lipolysis and lipogenesis, and the need for different lipids and cholesterol for adipocyte hypertrophy could explain lipoma formation,^12^ and can be associated with the changes in lipoprotein sub-fractions observed in Dercum’s disease.

The child maintains use of the current medication for pain relief; however, there is no evidence of pain reduction in the evolution of Dercum’s disease in adults, at least in studies reporting a five-year follow-up. This case remains a challenge for physicians, the patient, and her family, who face difficulties to restore a normal life. Future research is needed to detect the etiology and evolution of Dercum’s disease from childhood to adulthood.
